# COVID-19 Infection despite Previous Vaccination in Cancer Patients and Healthcare Workers: Results from a French Prospective Multicenter Cohort (PAPESCO-19)

**DOI:** 10.3390/cancers15194777

**Published:** 2023-09-28

**Authors:** Valérie Seegers, Guillaume Rousseau, Ke Zhou, Audrey Blanc-Lapierre, Frédéric Bigot, Hakim Mahammedi, Aurélien Lambert, Camille Moreau-Bachelard, Mario Campone, Thierry Conroy, Frédérique Penault-Llorca, Martine M. Bellanger, Jean-Luc Raoul

**Affiliations:** 1 Department of Biostatistics, Institut de Cancérologie de l’Ouest, 44805 Saint-Herblain, France; valerie.seegers@ico.unicancer.fr (V.S.); audrey.blanc-lapierre@ico.unicancer.fr (A.B.-L.); 2 Department of Biopathology, Institut de Cancérologie de l’Ouest, 49055 Angers, France; guillaume.rousseau@ico.unicancer.fr; 3 Department of Human and Social Sciences, Institut de Cancérologie de l’Ouest (ICO), 44805 Saint-Herblain, France; ke.zhou@ico.unicancer.fr (K.Z.); martine.bellanger@ico.unicancer.fr (M.M.B.); 4 Department of Medical Oncology, Institut de Cancérologie de l’Ouest, 49055 Angers, France; frederic.bigot@ico.unicancer.fr; 5 Department of Medical Oncology, Centre Jean Perrin, 63011 Clermont-Ferrand, France; hakim.mahammedi@clermont.unicancer.fr; 6 Department of Medical Oncology, Institut de Cancérologie de Lorraine, 54511 Vandoeuvre-lès-Nancy, France; a.lambert@nancy.unicancer.fr (A.L.); t.conroy@nancy.unicancer.fr (T.C.); 7 Department of Medical Oncology, Institut de Cancérologie de l’Ouest, 44805 Saint-Herblain, France; camille.moreau-bachelard@ico.unicancer.fr (C.M.-B.); mario.campone@ico.unicancer.fr (M.C.); 8 Department of Biopathology, Centre Jean Perrin, 63011 Clermont-Ferrand, France; frederique.penault-llorca@clermont.unicancer.fr; 9 Department of Social Sciences, EHEPS School of Public Health, 35043 Rennes, France; 10 Department of Clinical Research, Institut de Cancérologie de l’Ouest, 44805 Saint-Herblain, France

**Keywords:** COVID-19 vaccine, cancer patients, healthcare workers, France, breakthrough infections

## Abstract

**Simple Summary:**

In two cohorts of vaccinated cancer patients and healthcare workers, 5% had COVID-19 infection after vaccination. These infections occurred more frequently in younger cancer patients with gastrointestinal cancer, gynecological or breast cancer, or a localized cancer and in patients receiving chemotherapy or targeted therapy when vaccinated. In both cohorts, these breakthrough infections occurred early after initiation of vaccination (Alpha SARS-CoV-2 variant) or several months after the end of vaccination (Omicron SARS-CoV-2 variant). In both cohorts these COVID-19 cases in vaccinated individuals were not severe, with only four cancer patients requiring oxygen therapy.

**Abstract:**

In a multicenter prospective cohort of cancer patients (CP; n = 840) and healthcare workers (HCWs; n = 935) vaccinated against COVID-19, we noticed the following: i/after vaccination, 4.4% of HCWs and 5.8% of CP were infected; ii/no characteristic was associated with post-vaccine COVID-19 infections among HCWs; iii/CP who developed infections were younger, more frequently women (NS), more frequently had gastrointestinal, gynecological, or breast cancer and a localized cancer stage; iv/CP vaccinated while receiving chemotherapy or targeted therapy had (NS) more breakthrough infections after vaccination than those vaccinated after these treatments; the opposite was noted with radiotherapy, immunotherapy, or hormonotherapy; v/most COVID-19 infections occurred either during the Alpha wave (11/41 HCW, 20/49 CP), early after the first vaccination campaign started, or during the Omicron wave (21/41 HCW, 20/49 CP), more than 3 months after the second dose; vi/risk of infection was not associated with values of antibody titers; vii/the outcome of these COVID-19 infections after vaccination was not severe in all cases. To conclude, around 5% of our CPs or HCWs developed a COVID-19 infection despite previous vaccination. The outcome of these infections was not severe.

## 1. Introduction

The COVID-19 pandemic caused by severe acute respiratory syndrome coronavirus 2 (SARS-CoV-2) led to major global health and social issues, claiming millions of lives and deeply impacting economies, health, education, and business. This disruptive period showed that science delivers results [1]. SARS-CoV-2 vaccines (some using new technologies based on messenger RNA) were developed very quickly, with international and public–private scientific collaboration. This resulted in an unprecedented number of mass vaccination programs and, less than 6 months after the approval of the first vaccines, 3.4 billion doses of vaccine had been administered. Patients with cancer were considered to be at high risk of severe COVID-19 [2,3], and most guidelines recommended prevention measures and COVID-19 vaccination for this population, as well as for health care workers (HCW), after evidence of their efficacy and safety in cancer patients (CP) [2,4,5,6,7]. But the waning effectiveness over time, despite boosters, and the development of variants of concern, particularly the Omicron variant, were an issue, and breakthrough infections in vaccinated individuals were reported [8,9,10,11].

In June 2020, we initiated the PAPESCO-19 study, analyzing prospective data from cancer patients (CP) and HCWs in four French Comprehensive Cancer Centers. We showed that both populations (CPs and HCWs) had a similar prevalence of COVID-19 infection, noted the major diagnostic importance of anosmia and the high proportion of asymptomatic cases among CPs, and demonstrated that the seropositivity, high after the first vaccine among HCWs and low among CPs, was close to 100% after the second injection in both populations. Unfortunately, we, as others, observed some infections in vaccinated populations [12,13,14]. The aim of the present article is to describe these vaccine breakthrough infections in both cohorts. As it is now known that the protection given by vaccination decreased over time and differed depending on the variant of concern, our main motivations were to describe these breakthrough infections, particularly in CPs, taking into account the timing of occurrence (related to vaccination date and variant of concern), to describe the outcome of these infections and to look for a possible impact of any ongoing anticancer treatment.

## 2. Material and Methods

This section has been reported previously [12,13,14]. The PAPESCO-19 (Patients et Personnels de Santé des Centres de Lutte Contre le Cancer pendant l’épidémie de COVID-19) prospective multicenter cohorts study took place in 4 French Comprehensive Cancer Centers, located in Angers, Clermont-Ferrand, Nancy, and Nantes. This was a one-year study, with the first enrollment on 17 June 2020; we closed inclusions on 16 June 2021, resulting in 2304 participants, 1233 CPs and 1071 HCWs.

Participation in the study was proposed to CPs aged 18 years and over, irrespective of whether they had presented symptoms since the COVID-19 outbreak, attending the centers for active treatment or for follow-up (only if treatment had been stopped for more than 1 year) for a solid cancer (no hematologic disease). HCWs enrolled voluntarily after being informed of the study via the cancer center intranet. Participants were followed up every three months over a full year. All participants signed an informed consent form, and the study was conducted in accordance with the Declaration of Helsinki. The Ethics Committee (CPP-IDF VIII, Boulogne-Billancourt) approved the study (number 20.04.15) on 15 May 2020. This study was registered at ClinicalTrials.gov, Identifier: NCT04421625.

At baseline and quarterly, participants i/reported the presence or not of symptoms and of documented COVID-19 infection and the results of RT-PCR tests conducted independently of the study in the case of symptoms or contact; ii/reported data about vaccinations received (date, name); iii/CP reported cancer treatment received (chemotherapy, immunotherapy, tyrosine kinase inhibitors, hormones, or others); iv/all participants had blood sampling for a rapid diagnostic test (a lateral flow immunoassay (LFIA) NG-Test^®^), and aliquots were frozen and kept for antibody detection and measurement. Baseline demographic data, clinical details, and cancer history were recorded in electronic case report forms.

Serum from frozen aliquots was analyzed for antibodies against the spike protein (Liaison^®^ SARS-CoV-2 TrimericS IgG, DiaSorin, Saluggia, Italy) following the WHO’s recommendations (NIBSC 20/136) for standardizing analytical comparability. The detection threshold was 4.81 BAU/mL (Binding Antibody Units/mL), and the upper antibody titer limit was capped at 2080 BAU/mL.

In the present study, for participants who had received at least one SARS-CoV-2 vaccine injection, we compared the characteristics (at the time of the first vaccine injection) of those who reported a COVID-19 infection (breakthrough infection) during their follow-up versus those who did not. The median follow-up duration after the first vaccination was short and heterogeneous in both our cohorts as study inclusions stopped on 16 June 2021 (follow-up of the last patient included ended on 28 June 2022) while vaccination began in the first days of January 2021. This short median follow-up after vaccination, therefore, did not allow us to capture all infections after vaccination.

The vaccine recommendations were mRNA BNT162b2 (Pfizer) for CPs and HCWs over the age of 50 years, AZD1222 (AstraZeneca) for HCWs under 50 and the mRNA-1273 vaccine (Moderna) for some HCWs; although the mRNA BNT162b2 was soon used predominantly.

### Statistical Analysis

We conducted all analyses in the two cohorts separately; we did not perform any statistical comparisons between CPs and HCWs. For HCWs, we described age, sex, previous reported COVID-19 cases prior to the first vaccine injection, and antibody titration available in the following intervals: 0 to 3 months, 3 to 6 months, and over 6 months after vaccination. For CPs, we also described the cancer treatment history at the time of the first vaccine injection for surgery and systemic treatment with chemotherapy, immunotherapy, tyrosine kinase inhibitor, and hormonotherapy (none/ongoing treatment/stopped at the time of injection). The severity of infection was also documented (hospitalization requiring oxygen therapy, death) in self-reported questionnaires.

As the main outcome, we used the self-reported COVID-19 infection diagnosis, based on RT-PCR or antigenic test, reported quarterly by participants. We estimated the median follow-up using the inverse Kaplan–Meier method, using the time between the first vaccine injection and the last follow-up. For categorical and binary variables, we described them using number and percentage, and we compared them using Chi-square or Fisher’s exact tests, as appropriate. For quantitative variables, in the case of Gaussian distribution, we described characteristics using mean and standard deviation (SD), and we compared them using a Student *t*-test. For antibody titrations, due to the detection thresholds (<4.81 and >2080 BAU/mL), we considered them as censored quantitative variables, and we used median and range to summarize results and compared them using non-parametric rank tests. We did not impute missing data. All analyses were computed using R version 4.1.2 (R Core Team (2021)). R: A language and environment for statistical computing. R Foundation for Statistical Computing, Vienna, Austria. URL https://www.R-project.org/ (accessed on 12 November 2021).

## 3. Results

### 3.1. HCW

At least one vaccine injection was given to 935/1071 HCWs (87.3%) during the study period; their mean age was 42 years (SD: 10.2). The median follow-up after their first vaccination was 6.1 months (CI95%: 5.8–6.2 months). Before the first vaccine injection, 83 (8.9%) reported SARS-CoV-2 infections of which only one (1%) recurred after vaccination; 852 without previous infection received at least one injection; 40 (4.7%) had COVID-19 infection after being vaccinated (7 after one injection and 33 after a second). There was no statistical difference between infected (41, 4.4%) and non-infected (894, 96.0%) HCWs after vaccination regarding major clinical parameters (Table 1) including Body Mass Index (BMI) and diabetes. The proportion of infection in vaccinated HCWs was not related to the vaccine brand (*p* = 0.776): infection recurred in 16 of the 321 (5%) who received the AZ vaccine, 6/98 (6.1%) the Moderna vaccine, 0/3 the Janssen vaccine, and 19/485 (3.9%) the Pfizer vaccine; the vaccine name was not reported in 28 cases. Of the 935 HCWs, 5 (0.5%) were hospitalized (median length of stay: 1 day), all for a COVID-19 infection occurring before vaccination; none required oxygenotherapy; none were hospitalized in the ICU; and none died.

### 3.2. CPs

In the initial cohort of 1233 CPs, 50 were in follow-up and 1183 were receiving an active treatment at inclusion. At least one vaccination injection was given to 865 CPs including 25 under surveillance. In what follows, we focus on the 840 CPs vaccinated and receiving cancer treatment. The median follow-up after the first vaccination was 5.8 months (CI95%: 5.5–6.1); 625 (74.4%) were followed up 1 year as planned; 48 died before the end of follow-up (of the underlying disease); 131 decided to stop this follow-up without retracting their consent; 2 did not pursue the study due to medical decision; and 34 were lost to follow-up. The mean age of this population (n = 840) was 61.3 (SD 12.2) years. Women were younger than men, 59.4 years old (SD = 12.7) vs. 65.7 years old (SD = 10.1) (*p* < 0.0001). Data on tumor location, stage, treatment received before the date of the first vaccine injection in the whole population and in CPs who developed SARS-CoV-2 infection after vaccination are reported in Table 2.

In the cohort of 840 CPs, 793 (94.4%) did not report any previous COVID-19 infection before vaccination and 47 (5.6%) had had an infection before vaccination; in this latter group, 22 had received one vaccine injection and 25 had had 2 injections. After vaccination, 4 (8.5%) had a second COVID-19 infection, 1 after one vaccine injection, and 3 after two injections. In the 793 CPs who did not have COVID-19 infection before vaccination, 38 received one injection and 755 received two; 45 developed (5.7%) COVID-19 infection after vaccination, 11 after the first injection (5 later received a second injection), and 34 after two injections. Then, 49 CPs (5.8%) had COVID-19 infection despite vaccination (second infection in 4 cases and first in 45); this infection occurred within a median period of 268 days (extremes: 48–523) after the first vaccine injection. Regarding hospitalization in the 840 vaccinated CPs, 13 (1.5%) were hospitalized because of a COVID-19 infection, 4 required oxygenotherapy, none were admitted to an ICU, and none died. Of the 49 CPs who developed a COVID-19 infection after vaccination, 2 were hospitalized.

The 49 patients who developed an infection despite previous vaccination were significantly younger (55.2 vs. 61.7; *p* < 0.001) than those who did not. The infection was not significantly more frequently observed in women (7.0%) than in men (3.4%). These same infections were not-significantly more frequently observed in patients treated for gastrointestinal cancer (10.3%) or gynecological or breast cancer (6.9%) than patients with urologic cancer (2.1%). The cancer stage non-significantly influenced these infections that we observed in 4.2% of the patients with a metastatic stage, 6% of those with a locally advanced stage, and 8.6% of those with a localized stage. BMI and diabetes mellitus were not associated with SARS-CoV-2 infection after vaccination.

When we compare incidence of certain factors according to infection despite previous vaccination, following different therapeutic options (classified as follows: not received before vaccination, ongoing when vaccinated, received, and finished before vaccination), we observed:

- Non-significantly more infections in patients receiving or who did not receive systemic chemotherapy (6.7% and 8.1%), while those who had previously received systemic chemotherapy were less affected (4%);
- A significant difference (*p* = 0.004) regarding radiotherapy, with fewer infections in patients who were receiving or who had received radiotherapy (0% and 3%) compared to those who had not received radiotherapy (8.1%); in these analyses, we did not differentiate between those who had radiotherapy as their single treatment from those who received a radio-chemotherapy regimen (2 patients had an ongoing combination of chemotherapy and radiotherapy at the date of first vaccine injection);
- The same figures with hormonotherapy (*p* = 0.008) and immunotherapy (*p* = 0.014), with significantly more infection observed in patients who had not received the specific treatment (respectively, 7.3% and 7.6%) compared to those receiving these treatments at inclusion (0.7% and 2.4%) or who had finished the treatments (3.1% and 3.9%);
- No significant difference between groups in patients receiving targeted therapies;
- In addition, significantly we observed more infections in those who had never had cancer surgery (8.2%) compared to those who had been operated on (4.1%). However, in only 5.3% of patients who were operated on, surgery was performed less than 3 months after vaccination.

### 3.3. SARS-CoV-2 Infection and Epidemic Waves

**Figure 1:**
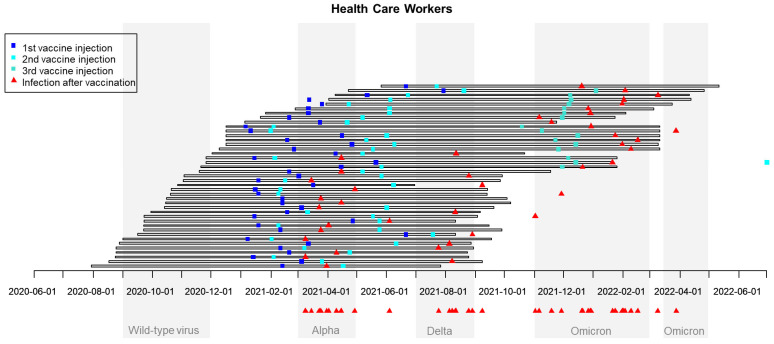
Chronological waves of SARS-CoV-2 infection in France and dates of vaccination and of breakthrough SARS-CoV-2 infections in HealthCare Workers (HCWs). The 41 HCWs with reported SARS-CoV infection despite vaccination are reported in 41 rows: horizontal bars correspond to the time between inclusion and the end of participation in the study, blue squares correspond to vaccine injections, and red triangles correspond to reported dates of COVID infection.

**Figure 2:**
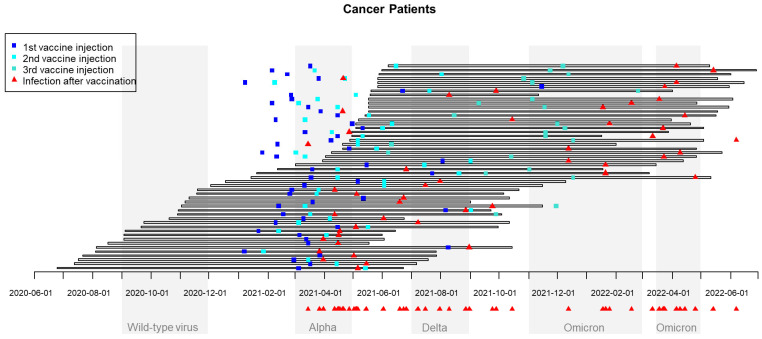
Chronological waves of SARS-CoV-2 infection in France and dates of vaccination and breakthrough SARS-CoV-2 infection in cancer patients. The 49 CPs with reported SARS-CoV are reported in 49 rows: horizontal bars correspond to the time between inclusion and the end of participation in the study, blue squares correspond to vaccine injections, and red triangles correspond to reported date of COVID infection.

In France, seven waves of SARS-CoV-2 infection were observed: March–May 2020 (wild-type virus), September–November 2020, March–April 2021 [variant of concern (VOC) Alpha], July–August 2021 (VOC Delta), November 2021–February 2022 (VOC Omicron), March–April 2022, and summer 2022 (both VOC Omicron). In the population studied of infected CPs despite previous vaccination, 20 cases occurred very early during the second quarter of 2021 (during the VOC Alpha wave), 9 cases during the second semester (6 during the VOC Delta wave), and 20 cases during the VOC Omicron wave (Figure 1). In the HCWs population, the temporal distribution was very similar: 12 cases during the first semester of 2021 (11 during the first quarter, VOC Alpha wave), 8 in the second semester of 2021 (7 during the VOC Delta wave), and 21 cases during the VOC Omicron wave (November 2021–February 2022) (Figure 2).

### 3.4. Antibody Titration in Vaccinated Participants

Regarding immune response to vaccination, median (and range) titers of SARS-CoV-2 antibodies in three periods (before the third month post-vaccination, between the third and sixth months, and after the sixth month) were similar between those who developed or not a post-vaccination infection in both populations of CPs and HCWs. (Table 3) As expected, median SARS-CoV-2-antibody titration values were higher between the third month and sixth month post vaccination and then decreased. We observed the same trend in both populations of CPs and HCWs. Of note, overall values were higher in HCWs except in the first quarter after vaccination.

## 4. Discussion

The PAPESCO study began in June 2020, less than 6 months after the first cases of COVID-19 were observed in France. The study was conducted prospectively and simultaneously in four French Comprehensive Cancer Centers located in three different geographic areas: Grand-Est (Nancy, the most severely affected French region), Auvergne-Rhône-Alpes (Clermont-Ferrand), and Pays de la Loire (Angers, Nantes, the least severely affected region). The first vaccines were approved in December 2020, after phase 3 in the general population, demonstrating efficacy at preventing COVID-19 illness, including severe disease.

In the randomized controlled trials demonstrating the efficacy and safety of SARS-CoV-2 vaccines, efficacy was impressive with mRNA-based vaccines (94.1%–95%) and those based on adenoviral vectors (between 62.1 and 90%) [15,16,17]. Most vaccine failures were observed early, either between the two doses [15] or soon after the second dose [17], but in these preliminary reports the median follow-up was short (2–3 months). A study assessed the durability of protection linked to the use of the mRNA-1273 SARS-CoV-2 vaccine in one of the seminal trials. The vaccine efficacy was of 92.6% at 40 days after dose 1, increased gradually to a peak of 94.1% at 120 days, then decreased to 89.6% at 200 days, showing that it was slightly waning [18]. Protection obtained from past infection against subsequent re-infection from the pre-Omicron variant was very high and long-lasting but not from the Omicron variant, but protection from severe disease was high for all variants [19].

In our population, most HCWs (935/1071: 87.3%) and CPs (865/1233: 70.2%) were vaccinated. In France, vaccination became mandatory for HCWs in September 2021, 3 months after the end of the follow-up for the first individuals included, and most HCWs were included very quickly, meaning that the vast majority of HCWs included were vaccinated during the study. The percentage of vaccinated HCWs was similar among our four Centers (between 86% and 92.9%). Regarding CPs, the vast majority were included while undergoing treatment and had metastatic or locally advanced disease, explaining why 200 died during the follow-up or stopped their follow-up prematurely. Nevertheless, more than two-thirds had been vaccinated. CPs who decided to stop their follow-up did so either because of a worsening of their disease or, conversely, because they had finished their treatment and begun their disease follow-up, which was mainly performed as remote monitoring.

In our core study population of individuals (HCWs, n = 935 and CPs undergoing cancer treatment, n = 840) who had received at least one vaccine dose, slightly more HCWs (8.8%) than CPs (5.6%) had had a SARS-CoV-2 infection before being vaccinated. Incidence of SARS-CoV-2 infection after vaccination was quite similar in both CP and HCW cohorts. Re-infection with SARS-CoV-2 in individuals who first had a COVID-19 infection then vaccination seemed more frequently observed in CPs (4 out of 50) than in HCWs (1/82).

In these special populations of CPs and HCWs developing an infection despite being vaccinated, the prognosis was excellent in HCWs and good in CPs, with only a few patients hospitalized, very few requiring oxygen, no hospitalizations in ICU, and no deaths. We, thus, confirm previous data showing that there was protection against severe infections. We did not analyze the incidence of long-COVID or COVID-19 sequelae and could not confirm in our small study group that, in CPs, immunization was also an effective measure for protecting patients from sequelae [20].

In our study, we could not find factors associated with the risk of vaccine failure in our HCWs population. Age was non-significantly lower in those who had an infection, but none of the other factors tested seemed of importance.

In contrast, we observed some factors associated or suggesting an association with a higher risk of vaccine failure in CPs. Younger CPs and women were more at risk, but CP women were significantly younger than CP men (59.4 ± 12.7 vs. 65.7 ± 10.1 years *p* < 0.0001). It is likely that most had breast cancer benefiting from adjuvant treatment and that they went back to “the normal world” during their follow-up (with more risks of contamination). BMI and diabetes were not associated with a higher risk. The cancer itself and its treatment had an impact. Patients treated for GI cancer (most often colon cancer) and gynecologic/breast cancer were slightly more at risk than patients with urologic cancer. Patients treated for cancer in a localized stage (i.e., receiving an adjuvant treatment) were (non-significantly) more at risk than those with locally advanced or metastatic disease, who certainly benefited from a longer duration of treatment. One possible explanation is that patients treated in an adjuvant setting for 3–6 months were adherent to preventive measures during this treatment but did not pay as much attention when back in normal life, with more contacts with friends and relatives. A work describing preventive behavior in PAPESCO CPs is ongoing, but for those receiving adjuvant treatment, preventive measures must be continued for a long time.

The impact of treatment is more difficult to assess. In the VOICE trial [4], 28 days after the second mRNA-1273 COVID-19 vaccination, while all control individuals could be considered to be protected, there were 7% of suboptimal or non-responders in CPs undergoing immunotherapy, 16% if treated with chemotherapy and 11% in those receiving the combination. An Italian prospective series enrolled patients under surveillance, chemotherapy, hormone therapy, targeted therapy, or immunotherapy to evaluate the seroconversion rate 21 days after the second dose of mRNA BNT162b2 vaccine. The lack of seroconversion was observed in 1.6% of those under surveillance, 13.9% on chemotherapy, 11.4% on hormone therapy, and 4.8% on immunotherapy [21]. Our data were reasonably similar, as the risk of infection despite vaccination was higher in those undergoing chemotherapy or targeted therapy when vaccinated than in those who had received this treatment before vaccination. In these patients, the vaccination needs to be conducted early, and preventive measures are of major importance. Some other studies show that risk factors for poor immune response were men, over the age of 65 years, and undergoing chemotherapy; CPs treated with immunotherapy had a better response [22] or confirmed that the seroconversion rate on immunotherapy was similar to that observed in controls and better than that observed on chemotherapy [23]. No clear difference was observed in our populations, but titration was performed at a fixed date from inclusion and not from vaccination; we thus had to analyze humoral response in quarters.

The timing of the occurrence of these infections despite previous vaccination was informative, showing a double impact of the time since vaccination and current variant of concern. In our experience, many infections were observed around April 2021, during the wave of the Alpha variant of concern (20 cases in CPs and 11 in HCWs) and, for many, between the first and second vaccine injections; high rates of infection were also noticed between November 2021 and April 2022, during the Omicron wave (20 cases in CPs and 21 in HCWs)—that is to say, quite far from the second injection for many and despite a booster injection. This is an argument in favor of booster injections not too far from the end of the usual vaccination schedule. In Norway, vaccine effectiveness was compared between HCWs and the general population taking the variant of concern into account. It was shown that this effectiveness was higher for the Delta variant than for the Omicron variant in both HCWs and non-HCWs. Surprisingly, vaccine effectiveness was higher among HCWs during the Omicron wave, perhaps linked to better prevention measures and routine testing [24]. A decrease in humoral response 6 months after the second dose of BNT162b2 was clearly demonstrated among vaccinated Israeli HCWs: the level of IgG antibodies decreased at a constant rate, whereas the neutralizing antibody level decreased rapidly for the first three months, with a slower decrease thereafter. This decrease was particularly clear in men, people over the age of 65 years, and people with immunosuppression. Surprisingly, obese participants had an increase in neutralizing antibody concentrations compared with non-obese participants [25].

We aim to share information from a large dedicated prospective multicenter cohort, which offers better evidence-based medicine findings than case-controlled or retrospective studies. This is the strength of the PAPESCO study. However, the evolving COVID-19 situation (epidemic waves and vaccine launch schedules) was unpredictable at the time of designing the study. Unfortunately, our follow-up was too short to have a broad overview of these breakthrough infections because the PAPESCO-19 study was designed for a duration of 2 years (12 months for inclusion and 12 months for individual follow-up). It is obvious that many infections may have been declared after the end of the follow-up and were not registered. Nevertheless, we believe that most of our results (outcome, impact of ongoing treatment and other predisposing factors) are true and remain pertinent.

## 5. Conclusions

To conclude, despite vaccination (and previous infection in 8.9% of HCWs and 5.6% of CPs), 4.4% of HCWs and 5.8% of CPs reported a COVID-19 infection with a median follow-up from vaccination of less than 6 months. The outcome of these infections in vaccinated individuals was in most cases not severe, with no deaths, no hospitalizations in the ICU, and a need for oxygen in only a few CPs. Only a few factors were associated with these infections after vaccination in CPs: young age, women, certain tumor locations (gastrointestinal cancers, gynecological and breast cancers), localized stages versus more advanced diseases, and certain treatments received while vaccinated; chemotherapy or targeted therapy during immunotherapy, hormonotherapy, and radiotherapy did not increase the risk.

Despite our short follow-up, we can thus suggest that vaccination prevents severe breakthrough infections in CPs, that a short delay between the first two injections is of importance, and that some tumor locations are more at risk, particularly patients receiving systemic chemotherapy or targeted treatments. Moreover, patients receiving adjuvant treatment must reinforce preventive measures after treatment.

## Figures and Tables

**Table 1:** Major clinical characteristics in Health Care Workers (HCWs) according to SARS-CoV-2 infection after vaccination.

		All HCWs (↨)	HCWs with Infection after Vaccination (⇥)	HCWs without Infection after Vaccination (⇥)	*p* Value
Total, N (%)		935	41 (4.4%)	894 (95.6%)	-
Age in years	mean (SD)	42 (10.2)	39.6 (9.7)	42.1 (10.3)	0.121
Sex, n (%)	women	758 (81.1%)	34 (4.5%)	724 (95.6%)	0.915
	men	177 (18.9%)	7 (4.0%)	170 (96%)	-
BMI, n (%)	<18 kg/m^2^	19 (2.1%)	1 (5.2%)	18 (94.7%)	0.351
	18–25 kg/m^2^	606 (66.4%)	31 (5.1%)	575 (94.9%)	-
	25–30 kg/m^2^	215 (23.5%)	7 (3.2%)	208 (96.7%)	-
	>30 kg/m^2^	73 (8%)	1 (1.3%)	72 (98.6%)	-
	missing	22	1	21	
Diabetes mellitus, n (%)	no	906 (99.1%)	35 (3.9%)	871 (96.1%)	0.276
	yes	8 (0.9%)	1 (12.5%)	7 (87.5%)	-
	missing	21	5	16	
Self-reported SARS-CoV-2 infection before the first vaccine injection, n (%)	none	852 (91.1%)	40 (4.7%)	812 (95.3%)	0.253
	at least one infection	83 (8.9%)	1 (1.2%)	82 (98.8%)	-

HCW: health care workers; SD: standard deviation; BMI: body mass index. Proportions are presented as column percentage (↨) for the whole HCW cohort and as row percentage (⇥) for subgroup values.

**Table 2:** Major clinical and therapeutic characteristics in CPs according to SARS-CoV-2 infection after vaccination.

		All Cancer Patients (↨)	CPs with Infection after Vaccination (⇥)	CPs without Infection after Vaccination (⇥)	*p* Value
Total, n		840	49 (5.8%)	791 (94.2%)	
Age in years, mean (SD)		61.3 (12.2)	55.2 (12.8)	61.7 (12.1)	0.001
Sex, n (%)	Women	573 (68.2%)	40 (7%)	533 (93%)	0.055
	Men	267 (31.8%)	9 (3.4%)	258 (96.6%)	-
BMI, n (%)	<18 kg/m^2^	16 (2.1%)	2 (12.5%)	14 (87.5%)	0.405
	18–24.9 kg/m^2^	357 (46.1%)	17 (4.8%)	340 (95.2%)	-
	25–30 kg/m^2^	258 (33.3%)	16 (6.2%)	242 (93.8%)	-
	>30 kg/m^2^	144 (18.6%)	7 (4.9%)	137 (95.1%)	-
	missing	65	7	58	
Diabetes mellitus, n (%)	No	578 (89.3%)	40 (6.9%)	538 (93.1%)	0.299
	Yes	69 (10.7%)	2 (2.9%)	67 (97.1%)	-
	missing	193	7	186	
Primitive tumor, n (%)	gastrointestinal cancer	29 (3.5%)	3 (10.3%)	26 (89.7%)	0.133
	gynecologic and breast cancer	479 (57%)	33 (6.9%)	446 (93.1%)	-
	urologic cancer	123 (14.6%)	3 (2.4%)	120 (97.6%)	-
	other location cancer	209 (24.9%)	10 (4.8%)	199 (95.2%)	-
Cancer stage, n (%)	localized stage	244 (29%)	21 (8.6%)	223 (91.4%)	0.064
	locally advanced stage	168 (20%)	10 (6%)	158 (94%)	-
	metastatic stage	428 (51%)	18 (4.2%)	410 (95.8%)	-
Self-reported SARS-CoV-2 infection before the first vaccine injection, n (%)	none	793 (94.4%)	45 (5.7%)	748 (94.3%)	0.346
	at least one infection	47 (5.6%)	4 (8.5%)	43 (91.5%)	-
Chemotherapy, n (%) ¥	not received	234 (27.9%)	19 (8.1%)	215 (91.9%)	0.09
	ongoing treatment	209 (24.9%)	14 (6.7%)	195 (93.3%)	-
	received and finished	396 (47.2%)	16 (4%)	380 (96%)	-
Radiotherapy, n (%)	not received	479 (57%)	39 (8.1%)	440 (91.9%)	0.004
	ongoing treatment	24 (2.9%)	0 (0%)	24 (100%)	-
	received and finished	337 (40.1%)	10 (3%)	327 (97%)	-
Hormonotherapy, n (%) §	not received	634 (75.8%)	46 (7.3%)	588 (92.7%)	0.008
	ongoing treatment	137 (16.4%)	1 (0.7%)	136 (99.3%)	-
	received and finished	65 (7.8%)	2 (3.1%)	63 (96.9%)	-
Immunotherapy, n (%) ¥	not received	541 (64.5%)	41 (7.6%)	500 (92.4%)	0.014
	ongoing treatment	247 (29.4%)	6 (2.4%)	241 (97.6%)	-
	received and finished	51 (6.1%)	2 (3.9%)	49 (96.1%)	-
Targeted therapy, n (%)	not received	536 (63.8%)	37 (6.9%)	499 (93.1%)	0.137
	ongoing treatment	208 (24.8%)	10 (4.8%)	198 (95.2%)	-
	received and finished	96 (11.4%)	2 (2.1%)	94 (97.9%)	-
Surgery, n (%)	no cancer surgery	353 (42%)	29 (8.2%)	324 (91.8%)	0.018
	surgery before vaccination:	487 (58%)	20 (4.1%)	467 (95.9%)	-
	<1 year	193/487 (39.6%)	9 (4.7%)	184 (95.3%)	0.789
	≥1 year	294/487 (60.4%)	11 (3.7%)	283 (96.3%)	-
	<3 months	26/487 (5.3%)	3/26 (11.5%)	23/26 (88.5%)	0.146
	>3 months	461/487 (94.7%)	17/461 (3.7%)	444/461 (96.3%)	-

CP: cancer patient; SD: standard deviation; BMI: body mass index. Proportions are presented as column percentage (↨) for the whole CP cohort and as row percentage (⇥) for subgroup values. ¥ 1 CP had missing information for the end date of this treatment; § 4 CPs had missing information for the end date of this treatment.

**Table 3:** Median and range of SARS-CoV-2 antibody titrations (in BAU/mL)/number of patients assessed, following the 1st vaccine injection. The results are presented for CPs and HCWs in relation to the occurrence of SARS-CoV-2 infection after vaccination during the follow-up.

Time from First Vaccine Injection to Titration	Population	Infection after Vaccination	No Infection after Vaccination	*p* Value
0 to 3 months	CP	551.5 (<4.81; >2080)/40	352.5 (<4.81; >2080)/620	0.605
0 to 3 months	HCW	384.5 (<4.81; >2080)/40	722 (<4.81; >2080)/837	0.402
3 to 6 months	CP	746.5 (<4.81; >2080)/20	631.5 (<4.81; >2080)/458	0.604
3 to 6 months	HCW	1935 (<4.81; >2080)/22	1310 (<4.81; >2080)/719	0.614
over 6 months	CP	402 (<4.81; >2080)/19	398 (<4.81; >2080)/321	0.414
over 6 months	HCW	992 (<4.81; >2080)/18	580.5 (12; >2080)/418	0.111

CP: cancer patients; HCW: health care workers.

## Data Availability

The data that support the findings of this study are available on request from one of the V.S.S.

## References

[1] Moeti M., Gao G.F., Herrman H. (2022). Global pandemic perspectives: Public health, mental health, and lessons for the future. Lancet.

[2] Lee L.Y.W., Cazier J.B., Starkey T., Briggs S.E.W., Arnold R., Bisht V., Booth S., Campton N.A., Cheng V.W.T., Collins G. (2020). COVID-19 prevalence and mortality in patients with cancer and the effect of primary tumour subtype and patient demographics: A prospective cohort study. Lancet Oncol..

[3] You B., Ravaud A., Canivet A., Ganem G., Giraud P., Guimbaud R., Kaluzinski L., Krakowski I., Mayeur D., Grellety T. (2020). The official French guidelines to protect patients with cancer against SARS-CoV-2 infection. Lancet Oncol..

[4] Oosting S.F., van der Veldt A.A.M., GeurtsvanKessel C.H., Fehrmann R.S.N., van Binnendijk R.S., Dingemans A.C., Smit E.F., Hiltermann T.J.N., den Hartog G., Jalving M. (2021). mRNA-1273 COVID-19 vaccination in patients receiving chemotherapy, immunotherapy, or chemoimmunotherapy for solid tumours: A prospective, multicentre, non-inferiority trial. Lancet Oncol..

[5] Massarweh A., Eliakim-Raz N., Stemmer A., Levy-Barda A., Yust-Katz S., Zer A., Benouaich-Amiel A., Ben-Zvi H., Moskovits N., Brenner B. (2021). Evaluation of Seropositivity Following BNT162b2 Messenger RNA Vaccination for SARS-CoV-2 in Patients Undergoing Treatment for Cancer. JAMA Oncol..

[6] Monin L., Laing A.G., Munoz-Ruiz M., McKenzie D.R., Del Molino Del Barrio I., Alaguthurai T., Domingo-Vila C., Hayday T.S., Graham C., Seow J. (2021). Safety and immunogenicity of one versus two doses of the COVID-19 vaccine BNT162b2 for patients with cancer: Interim analysis of a prospective observational study. Lancet Oncol..

[7] Fendler A., Shepherd S.T.C., Au L., Wilkinson K.A., Wu M., Byrne F., Cerrone M., Schmitt A.M., Joharatnam-Hogan N., Shum B. (2021). Adaptive immunity and neutralizing antibodies against SARS-CoV-2 variants of concern following vaccination in patients with cancer: The CAPTURE study. Nat. Cancer.

[8] Menni C., May A., Polidori L., Louca P., Wolf J., Capdevila J., Hu C., Ourselin S., Steves C.J., Valdes A.M. (2022). COVID-19 vaccine waning and effectiveness and side-effects of boosters: A prospective community study from the ZOE COVID Study. Lancet Infect. Dis..

[9] Shrotri M., Navaratnam A.M.D., Nguyen V., Byrne T., Geismar C., Fragaszy E., Beale S., Fong W.L.E., Patel P., Kovar J. (2021). Spike-antibody waning after second dose of BNT162b2 or ChAdOx1. Lancet.

[10] Bergwerk M., Gonen T., Lustig Y., Amit S., Lipsitch M., Cohen C., Mandelboim M., Levin E.G., Rubin C., Indenbaum V. (2021). Covid-19 Breakthrough Infections in Vaccinated Health Care Workers. N. Engl. J. Med..

[11] Andrews N., Stowe J., Kirsebom F., Toffa S., Rickeard T., Gallagher E., Gower C., Kall M., Groves N., O’Connell A.M. (2022). Covid-19 Vaccine Effectiveness against the Omicron (B.1.1.529) Variant. N. Engl. J. Med..

[12] Zhou K., Blanc-Lapierre A., Seegers V., Boisdron-Celle M., Bigot F., Bourdon M., Mahammedi H., Lambert A., Campone M., Conroy M. (2021). Anosmia but not ageusia as a COVID-19-related symptom among cancer patients. First results from the PAPESCO-19 cohort study. Cancers.

[13] Zhou K., Raoul J.L., Blanc-Lapierre A., Seegers V., Boisdron-Celle M., Bourdon M., Mahammedi H., Lambert A., Moreau-Bachelard C., Campone M. (2022). COVID-19 Infections in Cancer Patients Were Frequently Asymptomatic: Description From a French Prospective Multicenter Cohort (PAPESCO-19). Clin. Med. Insights Oncol..

[14] Seegers V., Rousseau G., Zhou K., Blanc-Lapierre A., Bigot F., Mahammedi H., Lambert A., Moreau-Bachelard C., Campone M., Conroy T. (2022). COVID-19 Vaccination Campaign in Cancer Patients and Healthcare Workers-Results from a French Prospective Multicenter Cohort (PAPESCO-19). Cancers.

[15] Polack F.P., Thomas S.J., Kitchin N., Absalon J., Gurtman A., Lockhart S., Perez J.L., Perez Marc G., Moreira E.D., Zerbini C. (2020). Safety and Efficacy of the BNT162b2 mRNA Covid-19 Vaccine. N. Engl. J. Med..

[16] Voysey M., Clemens S.A.C., Madhi S.A., Weckx L.Y., Folegatti P.M., Aley P.K., Angus B., Baillie V.L., Barnabas S.L., Bhorat Q.E. (2021). Safety and efficacy of the ChAdOx1 nCoV-19 vaccine (AZD1222) against SARS-CoV-2: An interim analysis of four randomised controlled trials in Brazil, South Africa, and the UK. Lancet.

[17] Baden L.R., El Sahly H.M., Essink B., Kotloff K., Frey S., Novak R., Diemert D., Spector S.A., Rouphael N., Creech C.B. (2021). Efficacy and Safety of the mRNA-1273 SARS-CoV-2 Vaccine. N. Engl. J. Med..

[18] Lin D.Y., Baden L.R., El Sahly H.M., Essink B., Neuzil K.M., Corey L., Miller J., COVE Study Group (2022). Durability of Protection Against Symptomatic COVID-19 Among Participants of the mRNA-1273 SARS-CoV-2 Vaccine Trial. JAMA Netw. Open.

[19] Team C.-F. (2023). Past SARS-CoV-2 infection protection against re-infection: A systematic review and meta-analysis. Lancet.

[20] Cortellini A., Tabernero J., Mukherjee U., Salazar R., Sureda A., Maluquer C., Ferrante D., Bower M., Sharkey R., Mirallas O. (2023). SARS-CoV-2 omicron (B.1.1.529)-related COVID-19 sequelae in vaccinated and unvaccinated patients with cancer: Results from the OnCovid registry. Lancet Oncol..

[21] Buttiron Webber T., Provinciali N., Musso M., Ugolini M., Boitano M., Clavarezza M., D’Amico M., Defferrari C., Gozza A., Briata I.M. (2021). Predictors of poor seroconversion and adverse events to SARS-CoV-2 mRNA BNT162b2 vaccine in cancer patients on active treatment. Eur. J. Cancer.

[22] Wankhede D., Grover S., Hofman P. (2023). Determinants of humoral immune response to SARS-CoV-2 vaccines in solid cancer patients: A systematic review and meta-analysis. Vaccine.

[23] Ruiz J.I., Lopez-Olivo M.A., Geng Y., Suarez-Almazor M.E. (2023). COVID-19 vaccination in patients with cancer receiving immune checkpoint inhibitors: A systematic review and meta-analysis. J. Immunother. Cancer.

[24] Langlete P., Tesli M., Veneti L., Starrfelt J., Elstrom P., Meijerink H. (2023). Estimated vaccine effectiveness against SARS-CoV-2 Delta and Omicron infections among health care workers and the general adult population in Norway, August 2021–January 2022. Vaccine.

[25] Levin E.G., Lustig Y., Cohen C., Fluss R., Indenbaum V., Amit S., Doolman R., Asraf K., Mendelson E., Ziv A. (2021). Waning Immune Humoral Response to BNT162b2 Covid-19 Vaccine over 6 Months. N. Engl. J. Med..

